# The relationship between secondary traumatic stress and secondary posttraumatic growth in clinical nurses: a cross-sectional study based on latent profile analysis

**DOI:** 10.3389/fpubh.2026.1909010

**Published:** 2026-10-02

**Authors:** Anni Hu, Ping Zhang, Yongpeng Dong, Juanhua Jia, Li Dong, Jiangying Han, Hongmei Zhu

**Affiliations:** 1 Department of Emergency, The First Affiliated Hospital of Anhui Medical University, Hefei, China; 2 Department of Cardiac Surgery, Nanjing Drum Tower Hospital, Affiliated Hospital of Medical School, Nanjing University, Nanjing, China; 3 Department of Nursing, The First Affiliated Hospital, Zhejiang University School of Medicine, Hangzhou, China; 4 Department of Nursing, The First Affiliated Hospital of Anhui Medical University, Hefei, China

**Keywords:** clinical nurses, cross-sectional study, latent profile analysis, secondary posttraumatic growth, secondary traumatic stress

## Abstract

**Objective:**

This study aims to identify the latent profiles of secondary traumatic stress-secondary posttraumatic growth (STS-SPTG) among clinical nurses and explore factors associated with profile membership.

**Methods:**

A convenience sampling method was used to recruit clinical nurses from a provincial tertiary Grade A hospital in Anhui Province between June and August 2024. Data were collected using the general information questionnaire, the Secondary Traumatic Stress Scale, the Secondary Posttraumatic Growth Inventory, the 10-item Big Five Inventory, and the Social Support Rating Scale. Latent profile analysis was performed to identify distinct STS-SPTG profiles among clinical nurses, and multinomial logistic regression analysis was used to explore factors associated with profile membership.

**Results:**

A total of 496 valid questionnaires were collected. Three latent profiles of STS-SPTG were identified among clinical nurses: the low-response profile (34.3%), the high-risk profile (42.3%), and the positive-growth profile (23.4%). Self-rated health status, job satisfaction, perceived job burnout, administrative position, personality traits, and social support were associated with latent profile membership among clinical nurses (*p* < 0.05).

**Conclusion:**

Clinical nurses exhibited substantial heterogeneity in STS-SPTG patterns. The identified profiles and associated factors may provide preliminary evidence for early identification and stratified supportive approaches for nurses with different psychological response patterns. However, associations involving personality traits should be interpreted cautiously and require further validation in future studies.

## 1 Introduction

Healthcare professionals who provide care for trauma survivors are repeatedly exposed to patients’ traumatic experiences, suffering, and intense negative emotions (1). Such indirect exposure may lead to dual psychological consequences, including secondary traumatic stress (STS) and secondary posttraumatic growth (SPTG), also referred to in some studies as vicarious posttraumatic growth (VPTG) (2, 3). Clinical nurses constitute a particularly important professional group in this context because they maintain continuous bedside contact with patients, provide both clinical care and emotional support, and are often required to respond empathically to patients’ pain, fear, grief, and uncertainty (4). Over time, sustained exposure to trauma-related narratives and emotional distress may affect nurses’ emotional well-being, cognitive appraisal, professional functioning, and quality of care (5).

STS refers to a set of trauma-like symptoms that arise from indirect exposure to others’ traumatic experiences through professional helping relationships. These symptoms commonly include intrusion, avoidance, and hyperarousal, which are similar to the core symptom clusters of posttraumatic stress reactions (6, 7). For nurses, STS may manifest as intrusive memories of patients’ suffering, emotional numbing, avoidance of trauma-related situations, sleep disturbance, irritability, and heightened vigilance (5). If not promptly identified and managed, STS may impair nurses’ psychological health, reduce job satisfaction and professional accomplishment, increase burnout and turnover intention, and ultimately threaten the quality and safety of nursing care (8–10).

In contrast, SPTG reflects the positive psychological changes that may occur after indirect exposure to trauma. Rather than simply representing the absence of distress, SPTG describes growth-related changes in self-perception, interpersonal relationships, professional competence, appreciation of life, spiritual experience, acceptance of others, and willingness to face new challenges (11, 12). For nurses, SPTG may contribute to improved professional identity, greater empathy, enhanced resilience, and better adaptation to demanding clinical environments (13, 14).

However, evidence regarding the relationship between STS and SPTG remains inconsistent. A study of oncology nurses reported that STS was negatively associated with VPTG, and that empathy mediated this relationship (4). Similarly, studies among newly graduated nurses and pediatric nurses suggested that compassion satisfaction, secondary traumatization, and meaning in work may shape the relationship between traumatic stress and psychological growth (15, 16). In contrast, research among emergency room physicians and nurses found that the association between STS and VPTG differed by professional role, suggesting that the relationship between negative and positive consequences of secondary trauma may vary across clinical contexts (17). These inconsistent findings indicate that STS and SPTG may not follow a simple linear pattern and that different subgroups of nurses may show distinct combinations of negative and positive trauma-related responses.

The coexistence of psychological distress and positive psychological transformation following indirect trauma exposure can be understood through the Conservation of Resources (COR) theory proposed by Hobfoll (18). COR theory suggests that individuals strive to acquire, maintain, and protect valued resources, including personal characteristics, social relationships, and other resources that support adaptation (19, 20). Exposure to patients’ traumatic experiences may threaten nurses’ existing resources, resulting in emotional exhaustion, psychological strain, and reduced professional efficacy, thereby increasing vulnerability to STS (21, 22). Conversely, when nurses can obtain or restore resources through effective coping processes and supportive interpersonal relationships, they may develop new perspectives, enhanced professional competence, and positive psychological changes, contributing to SPTG (21, 22). Therefore, COR theory provides the theoretical framework for understanding why nurses exposed to similar secondary trauma may experience different psychological outcomes. STS may reflect resource loss, whereas SPTG may represent resource restoration and growth after trauma exposure.

Accordingly, identifying heterogeneous psychological response patterns requires a person-centered analytical approach (23). Traditional variable-centered approaches primarily examine average associations among variables and assume relative homogeneity among individuals, which may overlook meaningful differences in nurses’ responses to secondary trauma (24). Latent profile analysis (LPA), a person-centered statistical method, can classify individuals into latent subgroups according to their response patterns across observed indicators. This approach helps maximize between-group heterogeneity and within-group homogeneity, thereby providing a more nuanced understanding of distinct psychological response patterns (25). In recent nursing research, LPA has been increasingly applied to identify latent profiles of emotional labor and occupational burnout among nurses, supporting the development of targeted interventions (26, 27).

Despite growing attention to STS and SPTG among nurses, few studies have examined their combined patterns using a person-centered approach. It remains unclear whether clinical nurses can be classified into distinct STS-SPTG profiles, such as high-risk profiles characterized by elevated STS and low SPTG, or adaptive profiles characterized by relatively low STS and high SPTG. Moreover, the factors associated with membership in different STS-SPTG profiles remain poorly understood.

Building on the COR theory framework described above, individual differences in trauma-related psychological responses may partly result from variations in internal and external resources (18, 20). Personality traits represent relatively stable internal resources that influence emotional regulation and responses to stressful experiences (28). The Big Five personality traits, including extraversion, openness, agreeableness, emotional stability, and conscientiousness, may contribute to individual differences in vulnerability to psychological distress and capacity for positive adaptation following secondary trauma exposure (29). Social support represents an important interpersonal resource that may reduce the negative effects of stress exposure and facilitate psychological adaptation (30). Therefore, personality traits and social support were examined in the present study as potential resource-related factors associated with STS-SPTG profile membership.

Although previous studies have explored factors associated with STS or SPTG separately (31, 32), limited evidence is available regarding whether these factors contribute differently to distinct combinations of STS and SPTG. Clarifying these associations may facilitate early identification of nurses at psychological risk and support the development of targeted, resource-oriented interventions.

Therefore, the present study aimed to identify latent profiles of STS and SPTG among clinical nurses using LPA and to examine whether individual, interpersonal and work-related factors were associated with profile memberships. By clarifying the heterogeneous patterns of negative and positive psychological responses to secondary trauma, this study may provide empirical evidence for early identification of high-risk nurses and for the development of precise, profile-specific interventions. These findings may also provide preliminary evidence for nursing managers to consider profile-specific supportive approaches aimed at promoting nurses’ psychological adjustment and maintaining occupational well-being.

## 2 Materials and methods

### 2.1 Study participants and procedure

A convenience sample of clinical nurses was recruited from a provincial tertiary Grade A hospital in Anhui Province, China, between June and August 2024. The inclusion criteria were: (1) registered nurses with a valid license, (2) currently engaged in clinical nursing practice, (3) having at least 1 year of clinical experience, and (4) voluntarily providing informed consent. Participants were excluded if they: (1) were visiting scholars or undergoing standardized training, (2) had experienced severe negative life events (e.g., bereavement, divorce, or accidents) within the past 6 months, (3) had a previously diagnosed severe physical or psychiatric disorder, or (4) were on sick or maternity leave during the survey period. All participants were employed full-time. Eligible nurses were invited to participate through the head nurses of each department. Because the survey link was distributed through the departments, the exact number of nurses who received the invitation and the number who declined participation were not recorded. The exclusion of nurses who had experienced severe negative life events within the previous 6 months was intended to minimize the potential confounding effects of concurrent personal trauma when examining STS and SPTG associated with indirect exposure to patients’ traumatic experiences. This study was approved by the Ethics Committee of the First Affiliated Hospital of Anhui Medical University (approval No. PJ2023-11-35).

### 2.2 Sample size consideration

A formal *a priori* power analysis was not performed because there is no universally accepted method for conventional power calculation in latent profile analysis (LPA) (25, 33). Methodological studies have indicated that sample size requirements for LPA are influenced by multiple factors, including the expected number of profiles, the degree of separation between profiles, the number of observed indicators, the proportion of the smallest profile, model complexity, and the amount of missing data (33–35). Previous simulation studies and methodological guidelines suggest that a sample size of approximately 500 can be adequate for identifying stable latent profiles in many applied research settings, provided that the smallest profile contains a sufficient number of participants (25, 34, 35). In the present study, 496 clinical nurses were included. In the final three-profile model, the smallest profile accounted for 23.4% of the sample (*n* = 116). Therefore, the adequacy of the sample for LPA was evaluated based on methodological recommendations, the total sample size, and the size of the identified profiles rather than a conventional power estimate.

### 2.3 Survey instruments

#### 2.3.1 General information questionnaire

A self-administered questionnaire was developed by the research team based on a literature review and expert discussion to collect participants’ background information. The variables were categorized into three main domains: (1) sociodemographic characteristics, including age, gender, place of origin (rural/urban), marital status, number of children, educational level, and average monthly household income; (2) work-related characteristics, comprising professional title, administrative position (defined as head nurse or deputy head nurse), department, years of work experience, employment type, average weekly working hours, and average number of night shifts per month; and (3) health status and specific experiences, including average daily sleep duration, self-rated health, participation in emergency rescue or training, and history of receiving professional psychological counseling. Additionally, specific psychosocial indicators, including perceived workload, job satisfaction, and perceived job burnout, were evaluated using single-item self-reported measures. These single-item measures were used to capture participants’ overall subjective perceptions of relevant occupational experiences and were considered exploratory indicators rather than comprehensive assessments of these multidimensional constructs. Similar approaches have been adopted in previous nursing studies using cross-sectional and latent profile analysis designs to characterize subjective occupational and psychosocial experiences (26, 36).

#### 2.3.2 Secondary traumatic stress scale (STSS)

The scale was developed by Bride et al. in 2004 and was translated and culturally adapted into Chinese by Li et al. in 2023 (6, 37). The scale is used to assess traumatic stress symptoms among professionals who provide services to trauma survivors. It consists of 17 items across three dimensions: Intrusion, Avoidance, and Arousal. Each item is rated on a 5-point Likert scale, ranging from 1 (never) to 5 (very often). The total score ranges from 17 to 85, with higher scores indicating more severe secondary traumatic stress. The Cronbach’s α coefficient in the present study was 0.921.

#### 2.3.3 Secondary posttraumatic growth inventory (SPTGI)

The scale was developed by Nina Ogińska-Bulik and colleagues in 2020 and was translated and culturally adapted into Chinese by our research team in 2023 (11, 38). The instrument is used to assess the degree of positive psychological changes experienced by professional helpers working with trauma survivors. The Chinese version consists of 12 items across four dimensions: Spiritual experience, Acceptance of others, Self-transformation, and New challenges. Each item is rated on a 6-point Likert scale, ranging from 0 (I have not experienced this change) to 5 (I have experienced this change to a very great degree). The total score ranges from 0 to 60, with higher scores indicating greater perceied positive change. In the present study, the overall Cronbach’s α coefficient of the scale was 0.936, and the Cronbach’s α coefficients for the four dimensions were 0.776, 0.785, 0.788, and 0.751, respectively.

#### 2.3.4 Ten-item personality inventory (TIPI)

The scale was developed by Gosling et al. in 2003 and was translated and culturally adapted into Chinese by Li in 2013 (39, 40). It is used to assess the five personality traits of the Big Five model. The Chinese version of the 10-Item Personality Inventory (TIPI-C) consists of 10 items across five dimensions: Extraversion (items 1 and 6), Agreeableness (items 2 and 7), Conscientiousness (items 3 and 8), Emotional stability (items 4 and 9), and Openness (items 5 and 10). Each item is rated on a 7-point Likert scale, ranging from 1 (strongly disagree) to 7 (strongly agree). Items 2, 4, 6, 8, and 10 are reverse scored. In the present study, the Cronbach’s α coefficients for the five dimensions were 0.728, 0.503, 0.631, 0.631, and 0.632, respectively. Because each TIPI-C dimension is assessed using only two items, relatively lower internal consistency is expected compared with longer personality inventories. Previous validation studies of both the original TIPI (39) and the Chinese TIPI-C (40) have similarly reported modest Cronbach’s α values, while demonstrating acceptable construct validity and test–retest reliability. Therefore, the reliability estimates in the present study were interpreted with caution.

#### 2.3.5 Social support rating scale (SSRS)

The scale was developed by Xiao in 1986 and revised in 1990 (41). The scale consists of 10 items across three dimensions: Objective support (items 2, 6, 7); Subjective support (items 1 and 3–5); and Utilization of social support (items 8–10). For items 1–4 and 8–10, the response options are scored from 1 to 4. Item 5 includes four subitems, A, B, C, and D, each scored from 1 (none) to 4 (full support), and the item score is calculated as the sum of the four subitems. For items 6 and 7, a response of “no source” is scored as 0; otherwise, the score is determined by the number of support sources selected. Higher total scores indicate greater overall social support. In the present study, the Cronbach’s α coefficient of the scale was 0.817.

### 2.4 Data collection and quality control

Data were collected online using Wenjuanxing^1^, a web-based survey platform. A questionnaire link and QR code were generated through the platform and distributed with the approval and support of the hospital nursing department. The questionnaire link was first sent to the head nurses of each department, who then invited eligible clinical nurses to complete the survey. Participation was entirely voluntary, and the head nurses were not involved in questionnaire completion and had no access to individual responses or subsequent data analysis. Participants were informed that their responses were anonymous and confidential and would only be used for research purposes.

To ensure the quality of the online survey, standardized instructions were provided at the beginning of the questionnaire, including the purpose and significance of the study, instructions for completion, and assurances of anonymity and confidentiality. All items were required before submission. Each device, mobile phone number, and IP address was allowed to submit the questionnaire only once. Questionnaires could not be submitted until all required items had been completed. After data collection, responses were exported to Excel and independently checked by two researchers.

Consequently, no missing data were present in the final dataset. Quality screening was performed according to predefined criteria. The 300-s threshold was predefined based on the length of the questionnaire, which contained approximately 70 items, and previous survey research using less than 5 s per item as a criterion for identifying potentially invalid responses (42). A threshold of 300 s was adopted as a conservative screening criterion. Questionnaires were excluded if the completion time was less than 300 s, if participants showed invariant responding across scale items, or if obvious regular response patterns were identified. Among the 577 collected questionnaires, 81 failed the predefined quality-screening criteria, resulting in 496 valid questionnaires being retained for the final analysis. No cases were excluded due to extreme values or statistical outliers. Preliminary comparisons were conducted between excluded participants (*n* = 81) and retained participants (*n* = 496) to assess potential selection bias due to data screening. The results showed that excluded participants were younger and had a higher proportion of male nurses. These differences were considered when interpreting the representativeness and generalizability of the findings.

### 2.5 Statistical analysis

Latent profile analysis (LPA) was performed using Mplus 8.3 software. Model fit was evaluated using the following indices: (1) the Akaike information criterion (AIC), Bayesian information criterion (BIC), and sample-size adjusted BIC (aBIC), with lower values indicating better model fit; (2) Entropy, ranging from 0 to 1, with values ≥ 0.80 indicating high classification accuracy; (3) the bootstrapped likelihood ratio test (BLRT) and the Lo–Mendell–Rubin adjusted likelihood ratio test (LMRT), for which *p* < 0.05 indicates that the *k*-class model fits significantly better than the *k*-1-class model. In the LPA models, the standardized scores of all 29 individual items from the STSS (17 items) and SPTGI (12 items) were used as continuous manifest indicators. Item-level indicators were selected to capture the multidimensional characteristics and heterogeneous response patterns of STS and SPTG. Before LPA, all item scores were transformed into Z-scores to ensure comparability across indicators, as the STSS and SPTGI use different response formats (5-point and 6-point Likert scales, respectively). For each latent profile solution, 200 random sets of starting values and 10 final-stage optimizations were specified to ensure that the maximum likelihood solution represented the best replicated solution rather than a local maximum. The optimal latent profile model was determined comprehensively based on model fit indices, model parsimony, and interpretability.

Data were analyzed using SPSS 27.0 software. Continuous variables with a normal distribution were presented as means and standard deviations, and between-group comparisons were conducted using one-way analysis of variance. Categorical variables were expressed as frequencies and percentages, and between-group comparisons were performed using the chi-square test. Before multinomial logistic regression analysis, multicollinearity among candidate predictors was assessed using tolerance values and variance inflation factors (VIF). No problematic multicollinearity was identified among the independent variables, with all tolerance values > 0.1 and VIF values < 5. Variables that showed statistically significant differences in univariate analyses were entered as independent variables, and the optimal LPA classification was used as the dependent variable. Multinomial logistic regression analysis was performed to identify factors associated with latent profile membership. A two-sided *p* < 0.05 was considered statistically significant.

## 3 Results

### 3.1 Characteristics of participants

A total of 577 questionnaires were collected, of which 496 met the predefined quality criteria and were included in the final analysis, yielding a valid questionnaire rate of 85.96%. The mean age of participants was 33.24 years (SD = 6.59; range, 20–59 years). The majority of participants were female (89.1%) and had a bachelor’s degree or above (92.3%). Regarding occupational characteristics, 43 (8.7%) nurses had ≤ 3 years of clinical work experience, whereas 453 (91.3%) nurses had >3 years of experience, and 54.1% held intermediate or senior professional titles. In addition, 29.0% of participants perceived their workload as heavy, 46.2% reported being satisfied with their current job, and 48.4% reported perceived job burnout. Detailed sociodemographic and work-related characteristics are summarized in Table 1.

**Table 1:** General information of clinical nurses and the univariate analysis of STS-SPTG potential profiles.

Variable	Total (*n* = 496, %)	Low-response group (*n* = 170, %)	High-risk group (*n* = 210, %)	Positive-growth group (*n* = 116, %)	*F*/*χ*^2^	*p*
Age (year, x̄ ± s)	33.24 ± 6.59	33.18 ± 6.75	32.76 ± 6.02	34.22 ± 7.28	1.869	0.155
Gender					1.137	0.566
Male	54 (10.9)	15 (8.8)	25 (11.9)	14 (12.1)		
Female	442 (89.1)	155 (91.2)	185 (88.1)	102 (87.9)		
Place of origin					0.321	0.852
Rural	323 (65.1)	112 (65.9)	138 (65.7)	73 (62.9)		
Urban	173 (34.9)	58 (34.1)	72 (34.3)	43 (37.1)		
Marital status					2.220	0.712
Married	360 (72.6)	125 (73.5)	148 (70.5)	87 (75.0)		
Single	128 (25.8)	43 (25.3)	59 (28.1)	26 (22.4)		
Others	8 (1.6)	2 (1.2)	3 (1.4)	3 (2.6)		
Number of children					9.765	0.045
0	164 (33.1)	63 (37.1)	70 (33.3)	31 (26.7)		
1	213 (42.9)	69 (40.6)	81 (38.6)	63 (54.3)		
≥2	119 (24.0)	38 (22.4)	59 (28.1)	22 (19.0)		
Education level					8.151	0.086
Associate degree	38 (7.7)	15 (8.8)	21 (10.0)	2 (1.7)		
Bachelor’s degree	443 (89.3)	149 (87.6)	184 (87.6)	110 (94.8)		
Master’s degree or higher	15 (3.0)	6 (3.5)	5 (2.4)	4 (3.4)		
Average monthly household income (CNY, yuan)					13.375	0.010
<5,000	76 (15.3)	24 (14.1)	32 (15.2)	20 (17.2)		
5,000–10,000	270 (54.4)	88 (51.8)	131 (62.4)	51 (44.0)		
>10,000	150 (30.2)	58 (34.1)	47 (22.4)	45 (38.8)		
Professional title					7.021	0.319
Nurse	42 (8.5)	15 (8.8)	20 (9.5)	7 (6.0)		
Senior nurse	186 (37.5)	65 (38.2)	73 (34.8)	48 (41.4)		
Nurse-in-charge	232 (46.8)	76 (44.7)	107 (51.0)	49 (42.2)		
Associate chief nurse and above	36 (7.3)	14 (8.2)	10 (4.8)	12 (10.3)		
Administrative position					12.099	0.002
No	435 (87.7)	153 (90.0)	191 (91.0)	91 (78.4)		
Yes	61 (12.3)	17 (10.0)	19 (9.0)	25 (21.6)		
Department					13.710	0.008
General wards	191 (38.5)	69 (40.6)	69 (32.9)	53 (45.7)		
Emergency and critical care	239 (48.2)	76 (44.7)	120 (57.1)	43 (37.1)		
Others	66 (13.3)	25 (14.7)	21 (10.0)	20 (17.2)		
Years of work experience (year)					0.242	0.886
≤3	43 (8.7)	16 (9.4)	18 (8.6)	9 (7.8)		
>3	453 (91.3)	154 (90.6)	192 (91.4)	107 (92.2)		
Average weekly working hours (h)					6.745	0.150
≤40	135 (27.2)	54 (31.8)	52 (24.8)	29 (25.0)		
41–50	298 (60.1)	94 (55.3)	137 (46.0)	67 (57.8)		
>50	63 (12.7)	22 (12.9)	21 (10.0)	20 (17.2)		
Average number of night shifts per month					6.660	0.155
≤5	193 (38.9)	72 (42.4)	72 (34.3)	49 (42.2)		
6–10	233 (47.0)	77 (45.3)	100 (47.6)	56 (48.3)		
>10	70 (14.1)	21 (12.4)	38 (18.1)	11 (9.5)		
Employment type					3.966	0.138
Long-term contract	408 (82.3)	146 (85.9)	173 (82.4)	89 (76.7)		
Permanent staff	88 (17.7)	24 (14.1)	37 (17.6)	27 (23.3)		
Average daily sleep duration (h)					6.779	0.034
≤7	201 (40.5)	59 (34.7)	99 (47.1)	43 (37.1)		
>7	295 (59.5)	111 (65.3)	111 (52.9)	73 (62.9)		
Participation in emergency rescue					0.689	0.709
No	306 (61.7)	109 (64.1)	126 (60.0)	71 (61.2)		
Yes	190 (38.3)	61 (35.9)	84 (40.0)	45 (38.8)		
Participation in emergency training					3.841	0.147
No	120 (24.2)	50 (29.4)	45 (21.4)	25 (21.6)		
Yes	376 (75.8)	120 (70.6)	165 (78.6)	91 (78.4)		
Received professional psychological counseling					1.765	0.414
No	448 (90.3)	151 (88.8)	194 (92.4)	103 (88.8)		
Yes	48 (9.7)	19 (11.2)	16 (7.6)	13 (11.2)		
Self-rated health					53.402	<0.001
Poor	19 (3.8)	3 (1.8)	13 (6.2)	3 (2.6)		
Fair	293 (59.1)	92 (54.1)	155 (73.8)	46 (39.7)		
Good	184 (37.1)	75 (44.1)	42 (20.0)	67 (57.8)		
Perceived workload					27.218	<0.001
Moderate	352 (71.0)	137 (80.6)	123 (58.6)	92 (79.3)		
Heavy	144 (29.0)	33 (19.4)	87 (41.4)	24 (20.7)		
Job satisfaction					105.854	<0.001
Dissatisfied	20 (4.0)	3 (1.8)	16 (7.6)	1 (0.9)		
Neutral	247 (49.8)	70 (41.2)	150 (71.4)	27 (23.3)		
Satisfied	229 (46.2)	97 (57.1)	44 (21.0)	88 (75.9)		
Perceived job burnout					104.930	<0.001
No	256 (51.6)	109 (64.1)	54 (25.7)	93 (80.2)		
Yes	240 (48.4)	61 (35.9)	156 (74.3)	23 (19.8)		
Extraversion (x̄ ± s)	8.28 ± 2.31	8.39 ± 2.16	7.55 ± 2.10	9.44 ± 2.40	28.088	<0.001
Agreeableness (x̄ ± s)	10.38 ± 1.84	10.44 ± 1.73	9.71 ± 1.73	11.51 ± 1.62	41.513	<0.001
Conscientiousness (x̄ ± s)	9.70 ± 2.07	9.84 ± 1.88	8.84 ± 1.82	11.07 ± 1.99	53.115	<0.001
Emotional stability (x̄ ± s)	9.08 ± 2.09	9.49 ± 1.76	7.89 ± 1.66	10.64 ± 1.98	96.432	<0.001
Openness (x̄ ± s)	8.49 ± 1.96	8.46 ± 1.92	7.88 ± 1.59	9.63 ± 2.13	33.591	<0.001
SSRS (x̄ ± s)	40.09 ± 8.93	41.05 ± 8.01	37.16 ± 8.76	43.98 ± 8.76	25.647	<0.001

SSRS, Social Support Rating Scale.

### 3.2 Descriptive analysis of STS and SPTG

Among the 496 clinical nurses, the mean scores for the STSS and the SPTGI were 41.31 (SD = 9.67) and 36.45 (SD = 11.79), respectively, indicating moderate levels of both secondary traumatic stress and secondary posttraumatic growth. The scores for each dimension of the two scales are presented in Table 2.

**Table 2:** Descriptive analysis of STS and SPTG.

Variable	Dimension	Minimum	Maximum	Mean	Standard deviation
STS	Total	17	82	41.31	9.67
	Intrusion	5	24	12.13	2.60
	Avoidance	7	33	16.83	4.66
	Arousal	5	25	12.36	3.30
SPTG	Total	0	60	36.45	11.79
	Spiritual experience	0	15	8.55	3.42
	Acceptance of others	0	15	9.06	3.21
	Self-transformation	0	15	9.89	3.09
	New challenges	0	15	8.95	3.03

STS, secondary traumatic stress; SPTG, secondary posttraumatic growth.

### 3.3 Latent profile analysis of STS-SPTG

Using the Z-scores of the 29 STSS and SPTGI items as manifest indicators, latent profile models with one to five profiles were fitted sequentially (Table 3). The results showed that the AIC, BIC, and aBIC values decreased gradually as the number of profiles increased. Compared with the two-profile model, the three-profile model showed substantially lower AIC, BIC, and aBIC values, a higher entropy value of 0.937, and significant BLRT and LMRT results, indicating good model fit and high classification accuracy. Although the four-profile model showed further decreases in AIC, BIC, and aBIC, a slight increase in entropy, and a significant LMRT result, the additional profile did not represent a theoretically distinct STS-SPTG response pattern. Instead, it primarily reflected a further subdivision of the low-response pattern identified in the three-profile model, with similarly low levels of both STS and SPTG across most indicators. In addition, the smallest profile accounted for only 5.6% of the sample, raising concerns regarding the stability and clinical interpretability of this additional classification. The five-profile model yielded a non-significant LMRT result and included a small profile that accounted for only 3.4% of the sample, suggesting potential over-extraction. Therefore, considering statistical fit indices, the substantive meaning of each profile, model parsimony, profile stability, and clinical interpretability, the three-profile model was selected as the optimal solution. In the three-profile model, the average posterior probabilities of class membership were 97.4%, 97.3%, and 97.2%, respectively, indicating high classification certainty.

**Table 3:** Fit indices of latent profile analysis.

Model	AIC	BIC	aBIC	Entropy	BLRT (*p*)	LMRT (*p*)	Category probability
1	40906.994	41150.976	40966.882	—	—	—	—
2	38337.072	38707.251	38427.936	0.927	<0.001	<0.001	0.546/0.454
3	37010.683	37507.059	37132.523	0.937	<0.001	<0.001	0.343/0.423/0.234
4	36022.573	36645.146	36175.390	0.952	<0.001	0.0405	0.056/0.373/0.200/0.371
5	35478.111	36226.882	35661.905	0.955	<0.001	0.1483	0.034/0.357/0.101/0.329/0.179

AIC, Akaike information criterion; BIC, Bayesian information criterion; aBIC, adjusted Bayesian information criterion; LMRT, Lo–Mendell–Rubin likelihood ratio test; BLRT, bootstrap likelihood ratio test.

The three latent profiles were named based on the distribution patterns of Z-scores across STSS and SPTGI items, with consideration of the overall psychological response characteristics and potential clinical implications of each profile (Figure 1). Class 1: low-response profile (*n* = 170, 34.3%), showed standardized scores that were generally below the mean across both STSS and SPTGI items. Nurses in this profile reported relatively low levels of secondary traumatic stress, together with limited perceived positive psychological change. Class 2: high-risk profile (*n* = 210, 42.3%), showed scores that were generally above the mean on STSS items and below the mean on SPTGI items. This pattern was characterized by a relatively high level of secondary traumatic stress, together with limited perceived positive psychological change. Class 3: positive-growth profile (*n* = 116, 23.4%), showed relatively low scores on STSS items and markedly higher scores on SPTGI items. This profile reflects a pattern of lower secondary traumatic stress accompanied by greater perceived positive psychological change.

**Figure 1:**
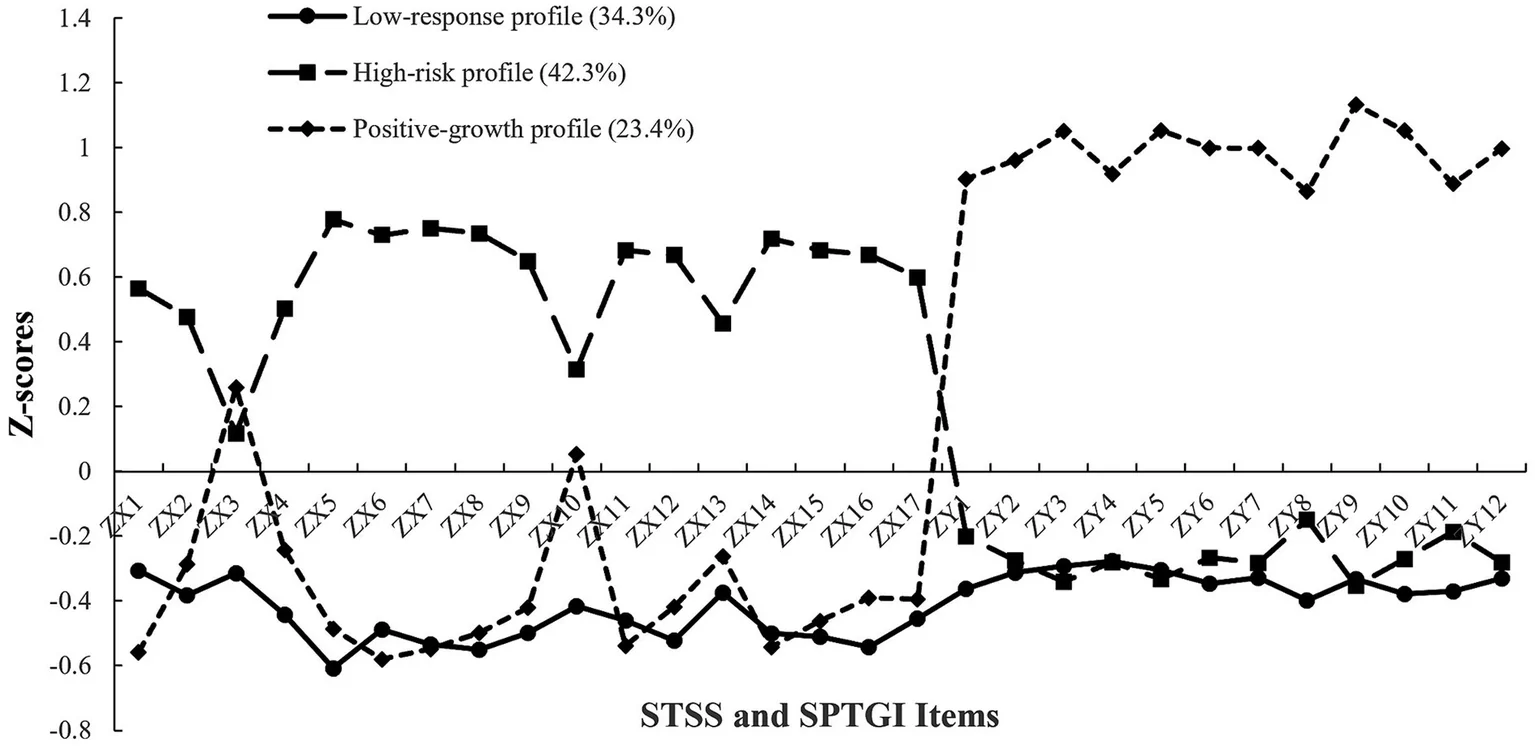
Latent profiles of STS-SPTG among clinical nurses. The x-axis represents the 17 items of the Secondary Traumatic Stress Scale (STSS) followed by the 12 items of the Secondary Posttraumatic Growth Inventory (SPTGI). The y-axis represents the standardized Z-scores of each item. The three lines represent the item-level response patterns of the low-response profile (34.3%), high-risk profile (42.3%), and positive-growth profile (23.4%). Different line types and point markers are used to distinguish the three profiles.

### 3.4 Univariate analysis of STS-SPTG latent profiles

Univariate analysis showed significant differences across the STS–SPTG latent profiles of clinical nurses in personality traits (extraversion, agreeableness, conscientiousness, emotional stability, and openness) and social support scores (all *p* < 0.001). Additionally, the latent profiles differed significantly in demographic and work-related characteristics, including the number of children, average monthly household income, administrative position, department, average daily sleep duration, self-rated health status, perceived workload, job satisfaction, and perceived job burnout (all *p* < 0.05). The results are summarized in Table 1.

### 3.5 Multinomial logistic regression analysis of STS–SPTG latent profiles

A multinomial logistic regression analysis was conducted using the three latent STS-SPTG profiles of clinical nurses as the dependent variable. Significant variables from the univariate analyses were included as independent variables, adjusting for continuous covariates.

The results indicated that, compared with the low-response profile, nurses with greater emotional stability and higher social support were associated with lower odds of membership in the high-risk profile (OR = 0.633, 95% CI: 0.527–0.760; OR = 0.954, 95% CI: 0.921–0.988). Conversely, fair self-rated health (vs. good), moderate job satisfaction (vs. satisfied), and reported perceived job burnout (vs. no perceived job burnout) were associated with higher odds of membership in the high-risk profile (OR = 1.947, 95% CI: 1.100–3.447; OR = 1.968, 95% CI: 1.097–3.529; OR = 2.130, 95% CI: 1.195–3.797).

Regarding the positive-growth profile, higher agreeableness was associated with higher odds of membership when compared with the low-response profile (OR = 1.243, 95% CI: 1.019–1.517). Additionally, nurses holding an administrative position (vs. no position) were also associated with higher odds of membership in this profile (OR = 2.663, 95% CI: 1.191–5.954). The results are summarized in Table 4.

**Table 4:** Multinomial logistic regression analysis of STS-SPTG latent profiles.

Variables	Classification	*β* value	Walds *χ*^2^	*p* value	OR (95% CI)
High-risk profile vs. low-response profile					
Intercept	-	6.092	16.251	<0.001	-
Emotional stability	-	−0.457	23.923	<0.001	0.633 (0.527, 0.760)
SSRS	-	−0.048	7.041	0.008	0.954 (0.921, 0.988)
Self-rated health (Good as a reference)	Poor	1.378	2.506	0.113	3.969 (0.720, 21.872)
	Fair	0.666	5.226	0.022	1.947 (1.100, 3.447)
Job satisfaction (Satisfied as a reference)	Dissatisfied	1.086	1.652	0.199	2.963 (0.565, 15.523)
	Neutral	0.677	5.155	0.023	1.968 (1.097, 3.529)
Perceived job burnout (No as a reference)	Yes	0.756	6.571	0.010	2.130 (1.195, 3.797)
Positive-growth profile vs. low-response profile					
Intercept	-	−6.242	15.614	<0.001	
Agreeableness	-	0.218	4.594	0.032	1.243 (1.019, 1.517)
Administrative position (No as a reference)	Yes	0.979	5.693	0.017	2.663 (1.191, 5.954)

SSRS, Social Support Rating Scale.

## 4 Discussion

### 4.1 Characteristics and theoretical interpretation of STS-SPTG profiles among clinical nurses

In the present study, the mean STSS score was 41.31 (SD = 9.67), indicating a moderate level of STS. This score was lower than the mean of 45.73 (SD = 14.53) reported among Chinese oncology nurses, whose STS level was classified as severe (4). The mean SPTGI score was 36.45 (SD = 11.79). According to the original SPTGI reference values, this score falls within the moderate range (11). It was slightly higher than the mean score of 33.90 (SD = 10.50) reported among nurses in the SPTGI development study (11). These differences may reflect variation in clinical settings, trauma exposure, and sample characteristics. However, the mean scores alone may not capture the heterogeneity of nurses’ psychological responses, which further supports the use of a person-centered approach in the present study.

This study identified three distinct latent profiles of STS-SPTG among clinical nurses: a low-response profile, a high-risk profile, and a positive-growth profile. The three-profile solution was preferred because it captured qualitatively distinct STS-SPTG configurations rather than additional subdivisions without clear theoretical meaning. These findings indicate that nurses exposed to similar indirect trauma experiences may develop different psychological response patterns, suggesting substantial heterogeneity in how individuals process and adapt to trauma-related occupational stress.

The identified profiles should also be interpreted within the cultural and healthcare context of China. Chinese nurses often work in a healthcare system characterized by high clinical demands, substantial patient-care responsibilities, and strong professional expectations regarding dedication and emotional commitment (43, 44). In addition, sociocultural values emphasizing professional responsibility, interpersonal harmony, and endurance may shape how nurses interpret occupational stress, regulate emotional responses, and utilize interpersonal resources (45, 46). Such contextual factors may influence the availability and utilization of psychological, interpersonal, and occupational resources, thereby shaping the manifestation and distribution of STS-SPTG profiles among Chinese clinical nurses.

However, the psychological processes reflected in these profiles may not be unique to the Chinese context. The coexistence of distress and positive psychological transformation has also been reported among nurses in different countries and clinical settings, suggesting that the identified profiles may represent a broader pattern of human adaptation to indirect trauma exposure, while their prevalence and specific characteristics may vary across cultural and healthcare environments. Similar person-centered findings have been reported among oncology nurses, underscoring the utility of latent profile analysis in delineating distinct patterns of psychological adaptation to indirect trauma (47). Moreover, existing evidence indicates that STS and vicarious posttraumatic growth are not simply opposite constructs; instead, they may coexist and show complex associations depending on individual and contextual resources (48).

Taken together, these findings can be interpreted within the COR framework introduced earlier (19, 20). Rather than acting independently, personal, interpersonal, and occupational resources may jointly shape how nurses respond to indirect trauma exposure (49). An imbalance between trauma-related demands and available resources may contribute to the high STS and low SPTG pattern, whereas greater access to and use of supportive resources may facilitate more adaptive responses (21, 22). Thus, the three profiles may reflect different patterns of resource availability and recovery rather than simply different levels of psychological symptoms.

The low-response profile accounted for 34.3% of the sample and was characterized by below-average levels of both STS and SPTG. Nurses in this subgroup did not exhibit prominent stress responses, yet they also reported limited positive psychological transformation. This indicates that the absence of severe psychological distress does not necessarily equate to adaptive growth. Posttraumatic growth is generally conceptualized as a complex process requiring cognitive processing, meaning reconstruction, and the mobilization of psychosocial resources, rather than an automatic byproduct of trauma exposure (50). Consequently, nurses in this profile may benefit from targeted interventions that facilitate reflective practice, professional meaning-making, and the positive reinterpretation of trauma-related clinical experiences.

The high-risk profile represented the largest subgroup (42.3%) and was defined by high STS and low SPTG. This pattern reveals that a considerable proportion of clinical nurses experience a substantial psychological burden without corresponding positive transformation. Repeated exposure to patient suffering, clinical deterioration, and death may be associated with intrusive symptoms, emotional exhaustion, and a diminished sense of professional meaning. The relatively high proportion of nurses classified into the high-risk profile should also be interpreted in light of the timing of data collection. Although the data were collected in 2024, most nurses in this study had more than 3 years of clinical experience (91.3%), suggesting that the majority may have been working during the COVID-19 pandemic and may have encountered pandemic-related occupational challenges. However, COVID-19-specific exposure was not assessed, and the contribution of pandemic-related experiences to the observed STS-SPTG patterns remains uncertain. Nevertheless, previous longitudinal studies have documented persistent psychological distress, traumatic stress, and emotional exhaustion among nurses and healthcare workers following prolonged pandemic-related occupational demands (51, 52). Therefore, the observed STS-SPTG patterns may reflect not only routine indirect trauma exposure but also accumulated occupational strain from the preceding years. Recent studies report a high prevalence of STS among emergency and oncology nurses, highlighting it as a critical occupational mental health concern (4, 53). Furthermore, evidence from newly graduated nurses suggests that a lack of positive professional experiences, such as compassion satisfaction, may impede the translation of traumatic stress into growth (15). Ultimately, when trauma-related demands exceed available coping resources, nurses may experience greater STS and reduced opportunities for positive cognitive restructuring.

The positive-growth profile (23.4%) was characterized by lower STS and markedly higher SPTG, reflecting a highly adaptive psychological response. These nurses may be better able to utilize available psychological and social resources to derive personal strength and professional meaning from trauma-care experiences. Previous research suggests that resilience, professional identity, social and organizational support, deliberate rumination, and adaptive emotion regulation are associated with posttraumatic growth among nurses (13, 54). Qualitative evidence further corroborates the view that PTG among critical care nurses is a dynamic trajectory of stress, adjustment, and growth rather than a spontaneous outcome (55). Importantly, these growth-oriented psychological resources have been shown to buffer against burnout and posttraumatic stress symptoms, suggesting a potentially protective role in sustaining nurses’ occupational mental health (56).

However, the interpretation of SPTG warrants caution. Although higher SPTG scores are often considered an indicator of positive psychological change, self-reported growth does not necessarily represent stable or objective transformation. Perceived growth may reflect both genuine psychological adaptation and cognitive processes through which individuals reconstruct meaning and cope with stressful experiences (57, 58). Therefore, the positive-growth profile identified in this study should be understood as a pattern of perceived psychological adaptation rather than definitive evidence of long-term personal transformation. Future longitudinal studies are needed to clarify whether these perceived changes persist over time.

### 4.2 High-risk profile: a resource-loss perspective

The factors associated with the high-risk profile are consistent with a resource-loss perspective. Within the COR framework, nurses in the high-risk profile may experience greater depletion of personal, interpersonal, and occupational resources, which reduces their ability to regulate distress and transform traumatic experiences into positive psychological change. Consistent with this resource-loss perspective, multinomial logistic regression analysis revealed that higher emotional stability and stronger social support were associated with lower odds of membership in the high-risk profile.

This finding suggests that individual psychological characteristics and interpersonal resources may be relevant to nurses’ psychological adaptation to trauma-related care. Specifically, higher emotional stability may be associated with more adaptive responses to trauma-related stress. However, given the measurement limitations of the TIPI-C, the observed association between emotional stability and high-risk profile membership should be considered exploratory. Previous research has similarly reported an association between neuroticism, the opposite pole of emotional stability, and posttraumatic stress symptoms among frontline nurses (59). Furthermore, social support may buffer the adverse effects of indirect trauma by providing emotional validation, practical assistance, and opportunities for shared reflection. Previous research aligns with this, demonstrating that social support not only mitigates STS but also partially mediates the relationship between STS and burnout in pandemic-care contexts (60, 61).

Conversely, fair self-rated health, moderate job satisfaction, and perceived job burnout were associated with increased odds of classification into the high-risk profile. These findings indicate that nurses with poorer perceived health and unfavorable work-related psychological states may have fewer available resources to cope with trauma-related stress. Self-rated health can serve as a broad indicator of perceived physical and psychological well-being; notably, recent longitudinal evidence highlights a bidirectional relationship between poor self-rated health and burnout symptoms (62). Thus, nurses reporting merely “fair” health may be more vulnerable to cumulative occupational stress and less equipped to mobilize adaptive coping mechanisms following trauma exposure.

The specific association between moderate job satisfaction and the high-risk profile highlights the potential importance of the work environment in relation to STS-SPTG profile membership. Job satisfaction may reflect nurses’ perceptions of professional value, organizational support, work environment quality, and sense of control. A recent meta-analysis of intensive care nurses confirmed a robust inverse association between job satisfaction and burnout, cementing its link to occupational mental health (63). Moreover, the association between perceived job burnout and the high-risk profile is consistent with meta-analytic findings of a strong positive correlation between STS and burnout among healthcare professionals (64). Burnout may be accompanied by depletion of emotional resources, diminished professional meaning, and reduced capacity for the cognitive processing involved in positive adaptation to traumatic experiences. However, because job satisfaction and perceived job burnout were assessed using single-item measures in the present study, these associations should be regarded as preliminary and hypothesis-generating rather than confirmatory.

Synthesizing these findings, membership in the high-risk profile appears to be associated not only with individual psychological characteristics but also with nurses’ physical health and occupational experiences. Given the exploratory nature of the personality findings, emotional stability should be regarded as a tentative correlate rather than a robust predictor of high-risk profile membership. Therefore, personality traits alone should not be used to identify nurses for targeted intervention. More broadly, nurses with limited social support may benefit from psychological and peer support, along with opportunities for structured reflection. For nurses reporting suboptimal health, lower job satisfaction, or burnout, future research may further examine their perceived work-related difficulties and access to appropriate psychological and occupational resources. Broader organizational strategies, such as workload management or improvement of the work environment, could be explored in future intervention studies. However, their effectiveness in reducing STS or enhancing SPTG was not evaluated in the present study.

### 4.3 Positive-growth profile: a resource-gain perspective

These findings suggest that positive-growth profile membership may be interpreted within a resource-gain framework, in which personal characteristics and occupational resources may be related to meaning reconstruction and adaptive responses after indirect trauma exposure. Within this framework, agreeableness may be considered a potential personal correlate, while administrative roles may reflect access to additional occupational resources. In the present study, higher agreeableness was associated with greater odds of membership in the positive-growth profile (relative to the low-response profile). Agreeableness is generally characterized by empathy, cooperativeness, interpersonal trust, and a prosocial orientation. In trauma-intensive clinical settings, these traits may be related to supportive interpersonal relationships and constructive engagement with challenging care experiences. However, given the limited internal consistency of the agreeableness dimension in the present sample, this association should be regarded as exploratory and interpreted cautiously. Recent study has reported positive associaton between personality traits, particularly agreeableness, and posttraumatic growth, with adaptive coping proposed as a possible explanatory pathway (65). Other research has slao reported an association between higher agreeableness and greater posttraumatic growth, which consequently fosters resilience (66). These findings suggest a possible relationship between agreeablenes, adaptive cognitive appraisal and meaning reconstruction.

Occupying an administrative position was also significantly associated with membership in the positive-growth profile. Nurses in managerial or administrative roles may have greater professional autonomy, involvement in decision-making, and access to organizational resources, which may enhance perceived control and professional meaning when coping with challenging work experiences. However, another possible explanation is that nurses in administrative positions may have less frequent direct contact with patients experiencing severe illness, suffering, or traumatic events compared with frontline clinical nurses. Reduced exposure to traumatic clinical situations may reduce vulnerability to STS and partly contribute to their classification into the positive-growth profile. Previous studies have shown that repeated exposure to patients’ traumatic experiences is an important occupational factor associated with secondary traumatic stress among nurses (60, 67). Therefore, the association between administrative positions and the positive-growth profile may reflect a combination of occupational resources and differences in trauma exposure rather than a specific advantage of managerial roles alone. This interpretation is also supported by previous studies suggesting that professional background, work environment, leadership support, social support, and adaptive coping are associated with posttraumatic growth among nurses (68–71).

Collectively, these findings suggest that future supportive approaches may focus on strengthening interpersonal and occupational resources, particularly for nurses with fewer available resources. Peer support, reflective practice, communication skills training, and team-based clinical debriefing may help facilitate adaptive meaning-making. The observed association with agreeableness should therefore be viewed as a preliminary finding that requires confirmation in future studies using more comprehensive personality measures. For frontline nurses facing challenging work experiences, additional occupational support may warrant further consideration and evaluation. However, specific organizational strategies, such as increasing participation in decision-making or strengthening leadership support, were not directly evaluated in this study and should therefore be regarded as broader practical implications rather than direct conclusions from the present study.

### 4.4 Limitations

The study has several limitations. First, although strict quality-control procedures were applied, differences in age, gender, and educational level were observed between excluded and retained participants. Therefore, potential selection bias from questionnaire screening cannot be completely ruled out, which may limit the generalizability of the findings. In addition, participants were recruited from a single hospital, which may further limit the representativeness of the sample. Future studies should include nurses from multiple hospitals and regions to improve the generalizability of the findings.

Second, although participation was voluntary and anonymous, questionnaire distribution involved head nurses as intermediaries, which may have introduced social desirability bias. More independent recruitment procedures should be considered in future studies to reduce potential response bias.

Third, although the TIPI-C provides an efficient assessment of the Big Five personality traits, each personality dimension is measured using only two items. Therefore, the internal consistency of individual dimensions may be relatively modest, thereby limiting the precision of personality assessment. Although previous studies have supported the validity of the TIPI-C, the associations between personality traits and STS-SPTG profile membership should be interpreted with caution. Future studies using more comprehensive personality inventories are warranted to validate these findings further.

Fourth, several occupational and psychosocial characteristics, including perceived workload, job satisfaction, and perceived job burnout, were assessed using single-item measures. Although these measures were effective at capturing participants’ overall subjective perceptions and reducing respondent burden in a large cross-sectional survey, they may not fully reflect the multidimensional characteristics of these constructs. Therefore, the associations between these indicators and STS-SPTG profile membership should be interpreted cautiously. Future studies should employ validated multi-item instruments to provide more comprehensive assessments of occupational and psychological factors.

Finally, participants who had experienced severe negative life events within the previous 6 months were excluded to reduce the potential confounding influence of concurrent personal trauma on psychological responses. However, this exclusion criterion may have restricted the range of psychological responses observed in the sample. Positive life events during the same period, such as the birth of a child, marriage, or promotion, were not assessed. These events may also have influenced STS or SPTG levels and therefore represent a potential confounding factor. Therefore, the identified STS-SPTG profiles may not fully reflect the diversity of psychological response patterns among clinical nurses. Future studies should consider both positive and negative life events and include broader samples. In addition, data were collected in 2024, and nurses’ psychological responses may still have been influenced by longer-term occupational strain related to the COVID-19 pandemic. As pandemic-related stress and cumulative occupational strain were not specifically assessed, the present study cannot determine the relative contributions of routine indirect trauma exposure and the longer-term consequences of the pandemic to the observed STS-SPTG patterns. These contextual factors should be considered when interpreting the findings and further examined in future longitudinal studies.

## 5 Conclusion

This study identified three heterogeneous STS-SPTG profiles among clinical nurses: the low-response profile, the high-risk profile, and the positive-growth profile. These findings suggest that nurses’ psychological responses to indirect trauma exposure are not uniform but vary substantially across individuals. The identified profiles provide further insight into the diversity of STS-SPTG responses and highlight the importance of considering individual differences in psychological adaptation. Personal, interpersonal, and occupational characteristics were associated with profile membership, although the personality-related findings should be regarded as preliminary. Recognizing nurses with high-risk psychological response patterns may facilitate timely assessment and further exploration of appropriate supportive interventions in future research. Although organizational-level approaches may have potential value, they were not evaluated in the present study, their effectiveness requires evaluation in future longitudinal or intervention studies.

## Data Availability

The raw data supporting the conclusions of this article will be made available by the authors, without undue reservation.

## Footnotes

1
www.wjx.cn
