# Supplementary material for: The relationship between secondary traumatic stress and secondary posttraumatic growth in clinical nurses: a cross-sectional study based on latent profile analysis
Source: Front Public Health. 2026 Oct 2;14:1909010. doi: 10.3389/fpubh.2026.1909010 (PMC13671999; doi:10.3389/fpubh.2026.1909010)
Supplement: Supplementary file 1 [file Table_1.DOCX]

Supplementary file

Supplementary Table S1. Complete multinomial logistic regression analysis of STS-SPTG latent profiles

| Variables | Classification | *β* value | Walds *χ*^2^ | *P* value | *OR*(95%*CI*) |
| --- | --- | --- | --- | --- | --- |
| High-risk profile vs.low-response profile | | | | | |
| Intercept | - | 6.092 | 16.251 | ＜0.001 | - |
| Extraversion | - | -0.035 | 0.265 | 0.607 | 0.966(0.846,1.103) |
| Agreeableness | - | -0.014 | 0.024 | 0.877 | 0.986(0.824,1.180) |
| Conscientiousness | - | -0.081 | 0.854 | 0.356 | 0.923(0.777,1.095) |
| Emotional stability | - | -0.457 | 23.923 | ＜0.001 | 0.633(0.527,0.760) |
| Openness | - | -0.051 | 0.353 | 0.552 | 0.950(0.802,1.125) |
| SSRS | - | -0.048 | 7.041 | 0.008 | 0.954(0.921,0.988) |
| Number of children (≥2 as a reference) | 0 | -0.598 | 2.218 | 0.136 | 0.550(0.250,1.208) |
|  | 1 | -0.243 | 0.528 | 0.467 | 0.784(0.408,1.510) |
| Average monthly household income(CNY,yuan) (＞10000 as a reference) | ＜5000 | -0.187 | 0.185 | 0.667 | 0.830(0.354,1.943) |
|  | 5000-10000 | 0.478 | 2.400 | 0.121 | 1.613(0.881,2.952) |
| Administrative position (No as a reference) | Yes | -0.024 | 0.003 | 0.956 | 0.976(0.412,2.311) |
| Department (Others as a reference) | General wards | 0.286 | 0.461 | 0.497 | 1.331(0.583,3.035) |
|  | Emergency and critical care | 0.515 | 1.600 | 0.206 | 1.674(0.753,3.721) |
| Average daily sleep duration(h)  (＞7 as a reference) | ≤7 | 0.253 | 0.896 | 0.344 | 1.288(0.763,2.173) |
| Self-rated health (Good as a reference) | Poor | 1.378 | 2.506 | 0.113 | 3.969(0.720,21.872) |
|  | Fair | 0.666 | 5.226 | 0.022 | 1.947(1.100,3.447) |
| Perceived workload (Heavy as a reference) | Moderate | -0.333 | 1.128 | 0.288 | 0.717(0.388,1.325) |
| Job satisfaction (Satisfied as a reference) | Dissatisfied | 1.086 | 1.652 | 0.199 | 2.963(0.565,15.523) |
|  | Neutral | 0.677 | 5.155 | 0.023 | 1.968(1.097,3.529) |
| Perceived job burnout (No as a reference) | Yes | 0.756 | 6.571 | 0.010 | 2.130(1.195,3.797) |
| Positive-growth profile vs.low-response profile | | | | | |
| Intercept | - | -6.242 | 15.614 | ＜0.001 |  |
| Extraversion | - | -0.007 | 0.010 | 0.920 | 0.993(0.871,1.133) |
| Agreeableness | - | 0.218 | 4.594 | 0.032 | 1.243(1.019,1.517) |
| Conscientiousness | - | 0.052 | 0.310 | 0.577 | 1.054(0.877,1.267) |
| Emotional stability | - | 0.172 | 3.079 | 0.079 | 1.187(0.980,1.438) |
| Openness | - | 0.158 | 3.390 | 0.066 | 1.171(0.990,1.385) |
| SSRS | - | 0.010 | 0.270 | 0.603 | 1.010(0.973,1.049) |
| Number of children (≥2 as a reference) | 0 | -0.327 | 0.510 | 0.475 | 0.721(0.294,1.769) |
|  | 1 | 0.252 | 0.484 | 0.487 | 1.286(0.633,2.613) |
| Average monthly household income(CNY,yuan) (＞10000 as a reference) | ＜5000 | 0.808 | 3.280 | 0.070 | 2.243(0.936,5.376) |
|  | 5000-10000 | 0.028 | 0.008 | 0.928 | 1.029(0.555,1.907) |
| Administrative position (No as a reference) | Yes | 0.979 | 5.693 | 0.017 | 2.663(1.191,5.954) |
| Department (Others as a reference) | General wards | 0.023 | 0.003 | 0.956 | 1.023(0.455,2.301) |
|  | Emergency and critical care | -0.469 | 1.278 | 0.258 | 0.626(0.278,1.410) |
| Average daily sleep duration(h)  (＞7 as a reference) | ≤7 | -0.003 | 0.000 | 0.992 | 0.997(0.561,1.771) |
| Self-rated health (Good as a reference) | Poor | -0.408 | 0.158 | 0.691 | 0.665(0.089,4.983) |
|  | Fair | -0.301 | 1.042 | 0.307 | 0.740(0.416,1.318) |
| Perceived workload (Heavy as a reference) | Moderate | -0.364 | 0.832 | 0.362 | 0.695(0.317,1.520) |
| Job satisfaction (Satisfied as a reference) | Dissatisfied | -0.635 | 0.233 | 0.629 | 0.530(0.040,6.970) |
|  | Neutral | -0.218 | 0.369 | 0.544 | 0.804(0.398,1.624) |
| Perceived job burnout (No as a reference) | Yes | -0.668 | 2.836 | 0.092 | 0.513(0.236,1.116) |

SSRS,Social Support Rating Scale
